# Effect of Cervical Manual Therapy on Sleep Quality: A Scoping Review of Randomized Controlled Trials

**DOI:** 10.3390/life15101557

**Published:** 2025-10-04

**Authors:** Do-Young Kim, Dong-Hyun Go, Hak-Jae Kim, Nam-Woo Lee, Yoon Jae Lee, Sook-Hyun Lee, In-Hyuk Ha

**Affiliations:** 1 Jaseng Korean Medicine Hospital, Seoul 06110, Republic of Korea; 2 Department of Public Health Science, Seoul National University, Seoul 08826, Republic of Korea; 3 Jaseng Spine and Joint Research Institute, Jaseng Medical Foundation, Seoul 06110, Republic of Korea

**Keywords:** cervical manual therapy, scoping review, sleep quality, randomized controlled trial, neuroendocrinology

## Abstract

Many individuals suffer from sleep disorders associated with chronic pain, neuroendocrine diseases, and primary sleep disorders. Although cervical manual therapy (CMT) is frequently presumed to enhance sleep quality in clinical settings, evidence regarding its effects on sleep remains inconclusive. We aimed to evaluate the therapeutic effect of CMT and clinical patterns, providing novel insights into its applicability for sleep disorders and further mechanism studies. Methods: A comprehensive literature survey was conducted by using 6 databases through February 2025, to identify randomized controlled trials (RCTs) assessing the effect of CMT on sleep quality as clinical outcome, regardless of primary diseases. Results: Among 1220 initial studies, a total of 10 RCTs involving 552 participants were included. All included RCTs assessed sleep quality using patient-reported outcome measures, while only one study utilized objective assessment via polysomnography. Among them, seven RCTs (70.0%) reported significant improvements in sleep quality that were not dependent on alleviating the primary diseases, with notable enhancements in subjective sleep depth and efficiency rather than sleep duration or latency. Sleep benefits were pronounced in primary sleep disorders, such as obstructive sleep apnea and bruxism, and in sleep disturbances secondary to other conditions, with limited effects in fibromyalgia (FM). Conclusions: With the dysregulated hypothalamic–pituitary–adrenal axis and aberrant serotonergic activity in FM, in this review, we formed a hypothesis and explored the potential effects of CMT on sleep-related serotonergic activity and HPA axis regulation. This scoping review underscores the need for further research to clarify the neuroendocrinological mechanisms underlying CMT’s role in sleep modulation and its potential applications in sleep-related disorders.

## 1. Background

Sleep is essential for systemic restoration and memory consolidation, and is regulated by complex neurochemical and endocrine mechanisms [1]. Poor sleep quality is associated with reduced quality of life (QoL) and increased risk of conditions such as cardiovascular disease, obesity, and mental illness [2,3]. According to the Centers for Disease Control and Prevention (CDC), 36.8% of US adults did not achieve sufficient sleep in 2022, and an estimated 50–70 million Americans suffer from chronic sleep disorders [4]. These disorders contribute to increased healthcare utilization, with an estimated annual economic burden exceeding USD 94.9 billion in the US [5].

The causes of sleep disorders are classified as either idiopathic, including restless legs syndrome, sleep apnea, and bruxism, or secondary to other conditions [6]. Underlying disorders associated with sleep disturbances include psychiatric conditions (e.g., depression and anxiety), pain-related disorders (e.g., chronic pain), and neuroendocrine disorders such as fibromyalgia (FM) and myalgic encephalomyelitis/chronic fatigue syndrome (ME/CFS) [7,8]. Treatment primarily targets the underlying pathology when identifiable, supplemented by pharmacologic therapy with central nervous system (CNS) agents and lifestyle interventions [9,10]. However, long-term use of psychotropic medications is discouraged due to concerns regarding efficacy and safety [11]. Additionally, sleep disturbances linked to conditions with unclear pathophysiology, such as FM and ME/CFS, highlight the need for novel therapeutic approaches for symptom management [12].

Cervical manual therapy (CMT), including spinal manipulation, chiropractic interventions, and massage, is widely utilized not only for pain relief and physical function improvement but also for mental health benefits, including stress reduction and sleep enhancement [13]. Mechanistically, CMT is suggested to improve sleep quality by modulating the autonomic nervous system (ANS) and neuroendocrine pathways, particularly the hypothalamic–pituitary–adrenal (HPA) axis with cortisol metabolism [14]. Additionally, evidence suggests that CMT may increase 5-hydroxytryptamine (5-HT) levels, a key neurotransmitter involved in sleep initiation and maintenance, indicating its potential utility in treating sleep disturbances [15,16]. However, its precise metabolic pathways within the CNS remain unclear, and high-quality clinical trials investigating its efficacy are limited [17,18].

Focusing on sleep quality, this study reviewed randomized controlled trials (RCTs) evaluating CMT with sleep quality as an outcome measure. By analyzing the characteristics and effects of CMT on both primary and secondary sleep disorders, this scoping review aims to evaluate its therapeutic effect and clinical patterns, providing novel insights into its applicability for sleep disorders and further mechanism studies.

## 2. Methods

This scoping review was conducted in accordance with the Preferred Reporting Items for Systematic Reviews and Meta-Analyses-Extension for Scoping Reviews (PRISMA-ScR) guidelines [19].

### 2.1. Research Questions of Scoping Review

Main question

- What is the current status of RCTs utilizing CMT that include sleep quality-related outcome measures?

Secondary questions

- What CMT techniques have demonstrated improvements in sleep quality?
- What are the characteristics of patient populations that showed or did not show improvement in sleep quality?
- In what aspects does sleep quality improve (e.g., sleep latency, duration, or depth)?
- Based on the above analyses, what directions should future research take to investigate the mechanisms underlying the effects of CMT on sleep quality?

### 2.2. Literature Search Strategy

A literature search was conducted using six electronic databases: PubMed, Cochrane Library, Embase, Medline, AMED, and Google Scholar, through February 2025. The primary search terms included “neck,” “cervical,” “spinal,” “manual therapy,” “spinal manipulation,” “chiropractic,” “sleep,” “sleep quality,” “insomnia,” “randomized controlled trial,” and “clinical trial.” Only RCTs were included, regardless of language. Detailed search strategies are provided in Supplementary Table S1.

### 2.3. Eligibility Criteria

Studies were included if they met the following criteria: (1) RCTs or randomized controlled crossover trials, (2) measurement of sleep-related symptoms as an outcome, (3) evaluation of efficacy of CMT compared to a control, and (4) availability of sleep-related outcome data with statistical analysis. Exclusion criteria included: (1) studies without full-text access, (2) participants are not with primary or secondary sleep disturbance, (3) trials comparing different types of manual therapies, and (4) studies not targeting the cervical spine (C1-C7 and C0 (occiput)) as one of the primary treatment regions.

### 2.4. Data Extraction

Extracted data included the number of participants, sex distribution, mean age, condition, type of CMT, treatment duration, session frequency, control intervention, and outcome measurement tools along with its overall results.

Due to heterogeneity of interventions regarding appellations and techniques, manual therapies were categorized into four types based on established terminology [20]: (1) joint manipulation (JM), including thrust and mobilization of joints; (2) soft tissue techniques (STT), involving muscle stretching and simple compression or friction of soft tissue; (3) myofascial techniques (MFT), including myofascial release and trigger point therapies; and (4) muscle energy techniques (MET), where patients contract muscles from specific positions in specific directions against the physician’s counterforce.

Treatment effectiveness was assessed using statistical comparisons with control groups. Intervention efficacy was classified as ‘Significant’ or ‘Not Significant (NS)’ based on the original study data, where ‘Significant’ indicated a statistical difference (*p* < 0.05) at the end of intervention treatment. ‘Partially significant’ was defined as a case in which only some of the multiple measurement outcomes assessing a single symptom domain in an RCT showed statistical significance.

### 2.5. Data Analysis

No additional statistical analysis was required. Participant numbers, age, treatment duration, and session frequency were reported as means and standard deviations (SDs).

## 3. Results

### 3.1. Characteristics of RCTs Meeting the Inclusion Criteria

**Figure 1:**
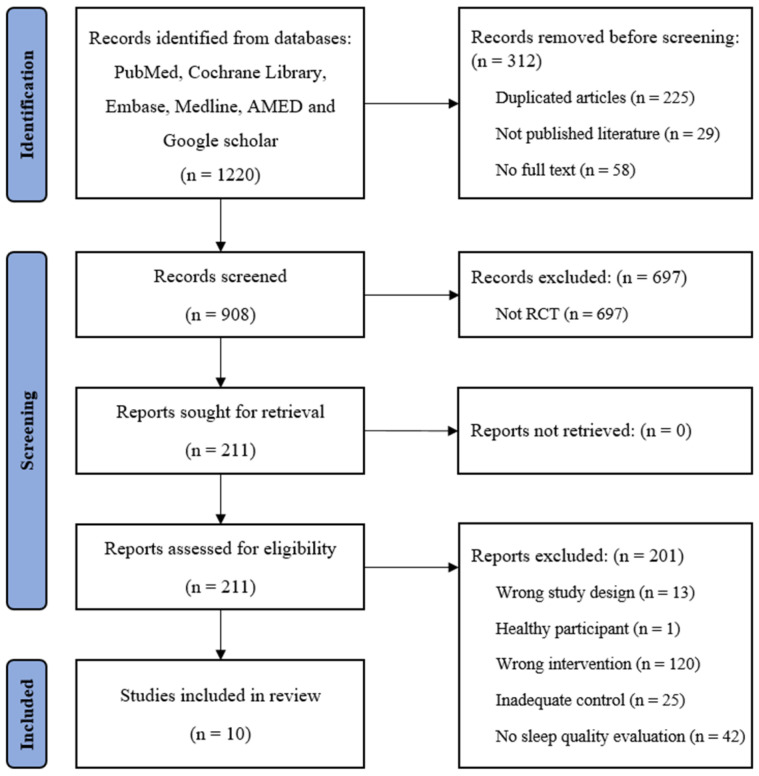
Flowchart of the study. CMT: cervical manual therapy; RCT: randomized controlled trial.

A total of 1220 articles were initially identified from the databases, and 10 RCTs met the inclusion criteria (Figure 1). These RCTs were conducted in eight countries, primarily in Europe (Spain: 2, Turkey: 2, Italy: 1, Poland: 1), with additional studies from Brazil, Egypt, India, and the US (1 RCT each). All articles were published after 2010, with seven published since 2020. The 10 included RCTs involved 552 participants (383 females) with a mean age of 49.6 ± 12.0 years, primarily diagnosed with FM (4 RCTs), chronic neck pain (2 RCTs), and sleep bruxism (2 RCTs) (Table 1).

### 3.2. Interventions and Outcome Measurements for Sleep Quality in RCTs

Among the total of ten RCTs, four utilized a single manual therapy technique, while the others employed multiple techniques. The most frequently used techniques were STT (7 RCTs, 70.0%), followed by JM and MTT (6 RCTs, 60.0% each), and MET (2 RCTs, 20.0%). The mean treatment duration was 51.9 ± 41.8 min per session, administered 11.6 ± 5.1 times over 5.6 ± 3.9 weeks (Table 1).

A total of 62 outcome domains were assessed (mean 6.2 ± 2.4 per RCT), with five RCTs incorporating at least one sleep-related primary outcome. Sleep quality assessments primarily relied on patient-reported measures, including the Pittsburgh Sleep Quality Index (PSQI, 9 RCTs), Epworth Sleepiness Scale (ESS, 2 RCTs), and Patient-Reported Outcomes Measurement Information System (PROMIS-sleep disturbance subscale, 1 RCT). Objective sleep parameters were assessed in single RCTs using polysomnography (PSG) (Table 1 and Table 2).

### 3.3. Effects on Sleep Quality in RCTs

Three RCTs focusing on sleep disorders enrolled patients with obstructive sleep apnea (OSA, 1 RCT) [21] and sleep bruxism (2 RCTs) [22,23]. All reported significant improvements in sleep quality, as measured by PSQI, compared to controls. However, findings on pain and temporomandibular joint (TMJ) function in bruxism patients were inconsistent. Additionally, no significant improvements in other sleep disorder-related parameters, such as bruxism and OSA symptoms, were observed.

Regarding other disorders, two RCTs on chronic neck pain [24,25] reported significant improvements in sleep quality, whereas three RCTs out of four on FM [26,27,28,29] showed non-significant benefit. Furthermore, except for one RCT demonstrating significant pain relief, all FM-related RCTs failed to improve FM symptoms, including fatigue, mental health, and pain threshold. One additional RCT involving post-cardiac surgery patients [30] reported partially significant improvements in sleep quality (Table 2).

### 3.4. Subscale Analysis

Three of the 10 RCTs included subscale analyses of sleep quality measurements. Except for one FM trial showing no significant improvements in any subscales, studies on chronic neck pain [25] and post-cardiac surgery [30] were included in the analysis. Notably, these studies commonly demonstrated improvements in subjective sleep quality, sleep efficiency, or sleep depth, whereas total sleep time, sleep latency, or sleep disturbance remained not significant (Table 2).

## 4. Discussion

CMT is widely used in clinical practice and is subjectively perceived by both practitioners and patients as beneficial for sleep quality. However, its underlying mechanisms and supporting evidence remain limited [31]. This review analyzed RCTs evaluating sleep quality, focusing on its characteristics in relation to the pathophysiology of the primary conditions.

### 4.1. Summary of Evidence

#### 4.1.1. Overall Efficacy of CMT on Sleep Quality

**Figure 2:**
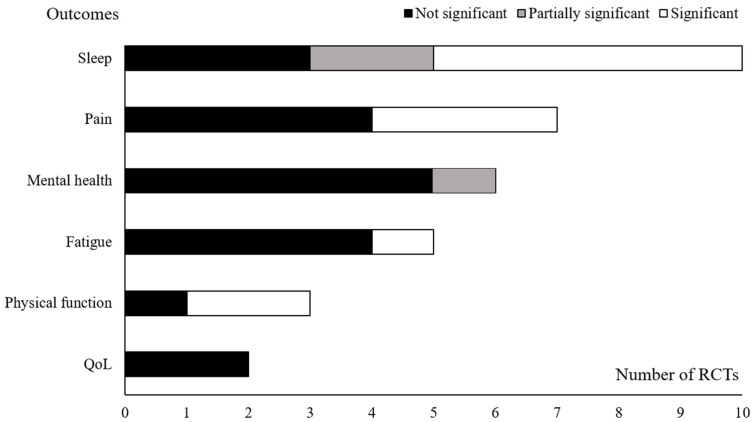
Graphical display for statistical significance of outcomes. ‘Significant’ indicates that the treatment demonstrated statistical significance (intervention vs. control, *p* < 0.05) based on the outcome measure at the predefined primary assessment time point. ‘Partially significant’ was defined as a case in which only some of the multiple measurement outcomes assessing a single symptom domain in an RCT showed statistical significance. Details of the symptom domains are as follows: Sleep (ESS, PROMIS, PSQI and PSG measurement), Pain (pain, pressure pain threshold), and Mental Health (anxiety, depression, emotional distress, and stress). For detailed information of these results, see Table 2. ESS: Epworth Sleepiness Scale, PROMIS: Patient Reported Outcomes Measurement Information System, PSG: polysomnography, PSQI: Pittsburgh Sleep Quality Index, QoL: quality of life, RCT: randomized controlled trial.

As shown in Figure 2, seven of 10 RCTs (70.0%) reported a statistically significant improvement in sleep quality compared to controls, on at least subset of measurements. Additionally, pain and physical function, the usual indications for manual therapy, improved in RCTs (42.9% and 66.7%, respectively), whereas other outcomes did not showed remarkable benefits [32,33]. Despite the heterogeneity of included conditions and main outcomes, these findings suggest a potential common effect of CMT on sleep quality that is not necessarily dependent on alleviation of primary disease (Table 2).

#### 4.1.2. Efficacy of CMT Across Conditions and Techniques

The pathophysiology of sleep disturbances varies across conditions [34]. In this study, chronic pain disorders were dominantly included, of which sleep impairment is primarily linked to HPA axis dysregulation as well as pain severity [35,36]. Regarding primary sleep disorders, the cause of OSA and sleep bruxism were poorly understood, although it was thought to be associated with stress and genetic predisposition [37,38] while post-surgical insomnia is influenced by environmental stress, anesthesia, and pain [39]. Results indicate that all studies investigating primary or secondary sleep disorders reported significant improvements in sleep quality, whereas notably lower improvement rates in FM (Table 2). CMT, known for its effects on pain relief and stress reduction, may primarily or adjunctively enhance sleep quality through pain modulation in chronic neck pain and autonomic regulation in conditions such as OSA and sleep bruxism. However, the limited efficacy observed in FM, characterized by both pain and autonomic dysfunction, suggests that the sleep-enhancing effects of CMT may not be solely attributable to these mechanisms. Although the range of conditions included in this review was limited, these findings imply the existence of a shared therapeutic pathway underlying CMT’s effects on sleep across different disorders, which is hardly applicable in the distinct pathophysiology of FM.

Regarding interventions, no consistent correlation was observed between intervention type or duration and sleep-related outcomes. Previous studies suggest that spinal manipulation for spinal alignment exerts effects independent of specific procedures or targeted lesions for pain perception [40,41]. This implies that functional connectivity within the cervical spinal complex as well as its overall alignment, along with brainstem interactions, may play a key role in modulating ascending neurotransmission pathways in the CNS [42].

#### 4.1.3. Effect on Sleep Depth, Efficiency, and Duration

Notably, despite the lack of applicable RCTs, subgroup analysis revealed that improvements in sleep quality were primarily observed in sleep depth and efficiency rather than sleep duration (Table 2). Deep sleep, or slow-wave sleep (SWS), is closely associated with subjective sleep quality and can be enhanced by non-pharmacologic interventions such as exercise, stress reduction, and light therapy [43,44,45]. These interventions are strongly linked to 5-HT modulation and HPA axis regulation. In this context, we focus on the potential effects of CMT on sleep quality, specifically through its influence on 5-HT and cortisol metabolism, considering the pathophysiology of the conditions examined in the included RCTs.

### 4.2. Modulating 5-HTergic Activity

Taken together, our results indicate that CMT improves sleep quality across heterogeneous conditions, excluding FM, possibly by enhancing sleep depth. To elucidate the shared underlying mechanisms, we conducted a scoping analysis focusing on serotonergic (5-HT) signaling and HPA axis regulation.

#### 4.2.1. Potential Linkage Between 5-HT, Sleep and CMT

5-HT, a melatonin precursor primarily secreted in the dorsal raphe nuclei in the brainstem, plays a crucial role in sleep maintenance as well as initiation [16]. The upper spinal cord is believed to be functionally linked to serotonergic activity in the brain and sleep regulation [46]. Studies on patients with cervical spinal cord injury (SCI) have reported poor sleep quality and significantly reduced nighttime melatonin levels, suggesting diminished 5-HTergic activity compared to both healthy individuals and those with thoracic SCI [47]. Regarding evidence from manual therapy, clinical studies on tuina, an Asian traditional manual therapy, have demonstrated increased peripheral 5-HT levels alongside improved sleep quality [15]. However, the relationship between peripheral 5-HT and central serotonergic activity remain unclear, and the poor methodological quality of these trials limits their interpretability.

5-HTergic hyperactivity in the CNS, which contributes to homeostatic sleep pressure, appears in various diseases with poor sleep quality, and decreases with efficient sleep [48,49]. Except for FM, our analysis indicates that CMT improved subjective sleep quality in both primary and secondary sleep disorders (Table 2). However, symptoms of sleep disorders such as sleep apnea and bruxism did not show improvement, implying independent sleep improvement effect of CMT. Dysregulation of 5-HTergic signaling during sleep has been implicated in the pathophysiology of phrenic nerve function and TMJ muscle activity, necessitating further research to clarify its role in symptom manifestation and subjective sleep quality [50,51]. Additionally, in patients with chronic neck pain, improvements in pain and physical function suggest that the role of CMT in 5-HT modulation-induced sleep improvement should be cautiously considered in interpretation.

#### 4.2.2. Pathologic Conditions of 5-HT Modulation and CMT

FM is characterized by reduced 5-HTergic activity within the CNS, with over 70% of patients experiencing prolonged fatigue and unrefreshing sleep [52]. These symptoms resemble those of ME/CFS, which typically showed reduction of 5-HT transporter [53]. Both FM and ME/CFS are considered neuroendocrinological disorders involving aberrant 5-HT and Kynurenine pathway activity, contributing to unresolvable fatigue despite sleep [54,55]. Although evidence linking spinal cord pathology to 5-HT metabolism in FM is limited, CMT may have had restricted effects due to abnormal 5-HT metabolism. Fragmented evidence from ME/CFS cases suggests that patients with cervical spinal stenosis experienced symptom resolution following surgery, indicating a potential organic spinal cord pathology [56]. However, further studies are required to elucidate the relationship between cervical spinal cord pathology and the pathophysiology of FM and ME/CFS.

### 4.3. Regulation of the HPA Axis

#### 4.3.1. Efficacy of CMT on HPA Axis and Sleep Architecture

Cortisol is a key regulator of the circadian rhythm and plays a critical role in stress response, energy metabolism, and immune function [57]. Elevated cortisol levels are commonly observed in various pathological states as part of the stress response, typically returning to baseline upon resolution of the primary condition [58]. A systematic review on the biochemical effects of spinal manipulation reported that cortisol and inflammatory regulation were influenced by the intervention [59]. Notably, CMT elicited a more pronounced cortisol response than thoracic manipulation, suggesting that HPA axis modulation may contribute to sleep improvement following cervical spinal cord intervention.

Cortisol also influences sleep architecture. Plasma cortisol levels decline during SWS, with SWS episodes significantly associated with this decrease [60]. SWS is reduced in individuals with sleep disorders and decline with aging [61]. During SWS, growth hormone secretion increases, promoting deep and restorative sleep [62]. Additionally, hypercortisolism disrupt AQP-4 function in the lymphatic system and perivascular circulation system within the brain, which contributes to poor subjective sleep quality [63]. Interestingly, a recent study identified that non-invasive manipulation of the cervical lymphatics, distributed in the soft tissues of the cervical spine, can increase cerebrospinal fluid drainage [64]. These findings suggest that CMT, especially targeting soft tissue rather than joint, may play a role in improving subjective sleep quality, as observed in RCTs, potentially by enhancing SWS.

#### 4.3.2. Dysregulation of HPA Axis in FM

However, in our data, RCTs particularly those involving FM participants, did not show improvements in mental health, fatigue-related outcomes, or sleep quality (Table 2). HPA axis dysregulation, characterized by adrenal insufficiency and cortisol depletion, is frequently reported in FM and ME/CFS [65]. Patients with FM exhibit reduced SWS compared to healthy individuals [52]. One RCT of FM in our study, which involved participants with a mean age below 40, demonstrated improved sleep quality following CMT, indicating ageing as a potential prognostic factor for HPA axis and SWS [26,66]. However, cortisol-targeted pharmacotherapies have failed to achieve symptom remission in FM and ME/CFS, underscoring the need to explore additional pathophysiological mechanisms related to HPA axis dysfunction [67,68].

### 4.4. Limitation and Perspectives

This scoping review has several limitations. First, the number of eligible RCTs was limited, and only half of those RCTs (5 out of 10) were considered to be of good quality, based on a PEDro score (0–10 points) of 6 or higher [69] (Supplementary Table S2). Also, considerable heterogeneity existed in terms of populations (demographic characteristics and clinical conditions) and treatment protocols (types of intervention and control). These factors hinder a comprehensive assessment of treatment effects on specific disorders and associated symptoms. Moreover, the available data were insufficient to analyze detailed sleep characteristics and patterns. Except for one RCT, none of the included studies employed objective sleep assessment tools such as PSG, rendering conclusions about sleep quality hypothetical.

Nevertheless, we pragmatically integrate the findings of published RCTs, focusing on the potential effects of CMT on sleep quality improvement. Further well-designed mechanistic studies and clinical trials are needed to refine our interpretations. Limited evidence exists on the relationship between CMT and spinal cord or brain activity in explaining sleep quality. Future studies should investigate the effects of CMT on sleep quality, particularly in the context of 5-HT and cortisol metabolism. Additionally, further studies are needed to quantitatively synthesize data from objective measures like PSG as well as subject survey outcomes, and determine which specific types of CMT are most effective for improving sleep efficiency, rather than duration. Also, along with pathophysiological challenges with FM, studies for neuroendocrinological effect of CMT regarding fatigue and mental health which are poorly alleviated in the RCTs are warranted.

## 5. Conclusions

This scoping review summarizes the current evidence from RCTs evaluating the effects of CMT that measured sleep quality-related outcome measures. While direct evidence remains scarce, we suggest the potential benefit of CMT in improving sleep quality via modulation of 5-HTergic activity within CNS and HPA axis. Given the scarcity of high-quality evidence, further RCTs with objective sleep assessments are warranted. Also, mechanistic studies exploring the role of CMT in neuroendocrine regulation may provide deeper insights into its therapeutic potential for sleep disturbance.

## Figures and Tables

**Table 1:** Study characteristics.

Items	Count
N. of RCT	10
N. of participants (female)	552 (383)
Mean N. of participant (±SD)	55.2 ± 30.1
Mean age (±SD) ^a^	49.6 ± 12.0
Condition of participants (N. of RCT, %)	10 (100.0)
Fibromyalgia	4 (40.0)
Chronic neck pain	2 (20.0)
Sleep bruxism	2 (20.0)
Obstructive sleep apnea	1 (10.0)
Post-cardiac surgery	1 (10.0)
Type of intervention (N. of RCTs, %) ^b^	10 (100.0)
Soft tissue technique (STT)	7 (70.0)
Joint manipulation (JM)	6 (60.0)
Myofascial technique (MFT)	6 (60.0)
Muscle energy technique (MET)	2 (20.0)
Mean treatment time per session (minutes ± SD)	51.9 ± 41.8
Mean N. of treatment time (±SD)	11.6 ± 5.1
Mean treatment period (weeks ± SD)	5.6 ± 3.9
Mean N. of measurements per RCT (±SD)	6.2 ± 2.4
Measurement for sleep quality (N. of RCTs, %) ^b^	
Pittsburgh Sleep Quality Index (PSQI)	9 (90.0)
Epworth Sleepiness Scale (ESS)	2 (20.0)
Patient Reported Outcomes Measurement Information System (PROMIS-sleep disturbance subscale)	1 (10.0)
Polysomnography (PSG)	1 (10.0)

^a^ This is the mean of ages presented as median or mean in original articles. ^b^ Some items have been applied multiple times in original articles; thus, the total percentage is larger than 100%. RCT: randomized controlled trial; SD: standard deviation.

**Table 2:** Summary of the RCTs for participants with physical disorders.

Disorder (Author, Year)	N. of Participants (Female, Mean Age)	Intervention (Control, Tx. Period)	Type of Tx. ^a^	Finding (Statistical Significance)	
				Subject Sleep Quality	Others
Primary Sleep Disorder					
Obstructive sleep apnea (Paolucci, 2023) [21]	52 (30, 61.0)	MFT ^d^ (myofunctional therapy, 4 weeks)	MFT	PSQI (total score): *p* < 0.01 ESS (total score): NS	snoring, SpO_2_: *p* < 0.05 AHI ^c^, N. of apneas, ODI, heart rate: NS
Sleep bruxism (Kadıoğlu, 2024) [22]	30 (21, 18–25 ^b^)	manual therapy (exercise, 8 weeks)	JM, MFT, STT	PSQI (total score) ^c^: *p* < 0.01	pain ^c^, stress ^c^, QoL ^c^, bruxism symptom, trigger point: NS
Sleep bruxism (Örenler, 2022) [23]	29 (29, 28.4)	manual therapy (occlusal splint, 8 weeks)	JM, MFT	PSQI (total score): *p* < 0.05	TMJ ROM, satisfaction: *p* < 0.05 pain, physical function: *p* < 0.01
Secondary Sleep Disorder					
Chronic neck pain (Cholewicki, 2022) [24]	87 (66, 42.1)	osteopathic manual therapy (wait-list, 4–6 weeks)	JM, MET, MFT, STT	PROMIS (sleep disturbance): *p* < 0.01	pain ^c^: *p* < 0.01 physical function ^c^, fatigue, depression: *p* < 0.05 activity, work, social role, anxiety: NS
Chronic neck pain (Hadamus, 2021) [25]	40 (32, 63.5)	MET ^d^ (Swedish massage, 2 weeks)	MET	PSQI (total score) ^c^: *p* < 0.01	-
Fibromyalgia (Ughreja, 2024) [26]	66 (58, 37.3)	craniosacral therapy (sham Tx., 12 weeks)	JM, MFT, STT	PSQI (total score) ^c^: *p* < 0.05	PPT, FM symptom, physical function, fatigue, kinesiophobia, emotional distress: NS
Fibromyalgia (Nadal-Nicolás, 2020) [27]	24 (24, 53.0)	massage (placebo, 4 weeks)	STT	PSQI (total score) ^c^: NS	pain ^c^: *p* < 0.05 fatigue ^c^, mood ^c^: NS
Fibromyalgia (Castro Sánchez, 2019) [28]	64 (58, 47.1)	MFT (dry needling, 4 weeks)	MFT, STT	PSQI (total score): NS	trigger pont ^c^, QoL, FM symptom, pain, anxiety, depression, fatigue: NS
Fibromyalgia (Moustafa, 2015) [29]	120 (52, 52.5)	manual therapy ^d^ (rehabilitative program 12 weeks)	JM	PSQI (total score): NS	spinal posture: *p* < 0.01 FM symptom ^c^, pain, PPT, anxiety, depression: NS
Post-cardiac surgery (Nerbass, 2010) [30]	40 (13, 61.9)	massage (no Tx., 3 days)	JM, STT	Subscales of PSQI, ESS sleep effectiveness ^c^: *p* < 0.05 total sleep time ^c^, sleep disorder ^c^: NS	fatigue, pain: NS

^a^ The interventions were categorized in five groups (JM: joint manipulation, MET: muscle energy technique, MFT: myofascial technique, STT: soft tissue technique). ^b^ This shows the range of age rather than mean age which is not available in original article. ^c^ This is primary outcome measurement. ^d^ This is additionally applied on control therapy, compared with control only. AHI: Apnoea/Hypopnoea Index, ESS: Epworth Sleepiness Scale, FM: fibromyalgia, NS: not significant, ODI: Oxygen Desaturation Index, PROMIS: Patient Reported Outcomes Measurement Information System, PPT: pressure pain threshold, PSQI: Pittsburgh Sleep Quality Index, QoL: quality of life, ROM: range of motion, TMJ: temporomandibular joint, Tx.: treatment.

## Data Availability

Data sharing is not applicable to this article as no datasets were generated and/or analyzed during the current study. Data visualized in the graphs of this manuscript are available from the corresponding authors upon reasonable request.

## Supplementary Materials

The following supporting information can be downloaded at https://www.mdpi.com/article/10.3390/life15101557/s1: Supplementary Table S1: Searching strategy for literature survey; Supplementary Table S2: Study quality of RCTs on the PEDro scale.

## List of Abbreviations

ANS	Autonomic nervous system
CMT	Cervical manual therapy
FM	Fibromyalgia
HPA	Hypothalamic–pituitary–adrenal axis
ME/CFS	Myalgic encephalomyelitis/chronic fatigue syndrome
MET	Muscle energy technique
MFT	Myofascial technique
RCTs	Randomized controlled trials
QoL	Quality of life
SCI	Spinal cord injury
STT	Soft tissue technique
5-HT	5-hydroxytryptamine
