# Supplementary material for: Effect of Cervical Manual Therapy on Sleep Quality: A Scoping Review of Randomized Controlled Trials
Source: Life (Basel). 2025 Oct 4;15(10):1557. doi: 10.3390/life15101557 (PMC12565680; doi:10.3390/life15101557)
Supplement: Supplementary file 1 [file life-15-01557-s001.zip › life-3883757-supplementary.pdf]

Supplementary Table S1. Searching strategy for literature survey

Search terms
Intervention 1 (OR)<br>“neck,” “head,” “occiput,” “cranial,” “cervical vertebrae,” “thoracic vertebrae,” “spinal,” “axial,” “cervical,” “thoracic,” “vertebra,” “vertebrae,” “vertebral,” “intervertebral.”
Intervention 2 (OR)<br>“manual therapy,” “spinal manipulation,” “chiropractic,” “manipulation,” “mobilization,” “thrust,” “craniosacral therapy,” “joint manipulation,” “soft tissue technique,” “myofascial technique,” “muscle energy technique,” “massage,” “manipulation,” “chuna,” “tuina,” “acupress,” “acupoint pressure.”
Outcome (OR)<br>“sleep quality,” “sleep,” “insomnia,” “sleep disorder,” “sleep latency,” “sleep efficiency”, “sleep architecture,” “REM sleep,” “Non-REM sleep,” “EEG,” “polysomnography,” “sleep diary.”
Study design (OR)<br>“randomized controlled trial,” “clinical trial,” “randomized,” “randomly,” and “trial.”
Search strategy: Intervention 1(AND) Intervention 2 (AND) Outcome (AND) Study design
Example of a full search string used in PubMed<br><br>(("neck"[MeSH Terms] OR "head"[MeSH Terms] OR "occiput"[MeSH Terms] OR "cranial"[MeSH Terms] OR "cervical vertebrae"[MeSH Terms] OR "thoracic vertebrae"[MeSH Terms] OR "spinal"[MeSH Terms] OR "axial"[MeSH Terms] OR "cervical"[MeSH Terms] OR "thoracic"[MeSH Terms] OR "vertebra"[MeSH Terms] OR "vertebrae"[MeSH Terms] OR "vertebral"[MeSH Terms] OR "intervertebral"[MeSH Terms] OR neck OR head OR occiput OR cranial OR cervical OR thoracic OR spinal OR axial OR vertebra* OR intervertebral)) AND (("manual therapy"[MeSH Terms] OR "spinal manipulation"[MeSH Terms] OR "chiropractic"[MeSH Terms] OR "manipulation"[MeSH Terms] OR "mobilization"[MeSH Terms] OR "thrust"[MeSH Terms] OR "craniosacral therapy"[MeSH Terms] OR "joint manipulation"[MeSH Terms] OR "soft tissue technique"[MeSH Terms] OR "myofascial technique"[MeSH Terms] OR "muscle energy technique"[MeSH Terms] OR "massage"[MeSH Terms] OR "chuna"[All Fields] OR "tuina"[All Fields] OR "acupress"[All Fields] OR "acupoint pressure"[All Fields] OR "manual therapy" OR "spinal manipulation" OR chiropractic OR manipulation OR mobilization OR thrust OR "craniosacral therapy" OR "joint manipulation" OR "soft tissue technique" OR "myofascial technique" OR "muscle energy technique" OR massage OR chuna OR tuina OR acupress* OR "acupoint pressure")) AND (("sleep quality"[MeSH Terms] OR "sleep"[MeSH Terms] OR "insomnia"[MeSH Terms] OR "sleep disorder"[MeSH Terms] OR "sleep latency"[MeSH Terms] OR "sleep efficiency"[MeSH Terms] OR "sleep architecture"[MeSH Terms] OR "REM sleep"[MeSH Terms] OR "Non-REM sleep"[MeSH Terms] OR "EEG"[MeSH Terms] OR "polysomnography"[MeSH Terms] OR "sleep diary"[MeSH Terms] OR "sleep quality" OR sleep OR insomnia OR "sleep disorder" OR "sleep latency" OR "sleep efficiency" OR "sleep architecture" OR "REM sleep" OR "Non-REM sleep" OR EEG OR polysomnography OR "sleep diary")) AND (("randomized controlled trial"[Publication Type] OR "clinical trial"[Publication Type] OR randomized OR randomly OR trial))

Supplementary Table S2. Study quality of RCTs on the PEDro scale

Author (year)	Random allocation	Concealed allocation	Baseline similarity	Subject blinding	Therapist blinding	Assessor blinding	<15% dropouts	Intention to treat analysis	Between-group difference reported	Point estimate, variability reported	Total (0–10)[a]
Kadıoğlu (2024)	Y	Y	Y	N	N	Y	Y	N	Y	Y	7
Ughreja (2024)	Y	Y	Y	N	N	N	N	N	Y	Y	5
Paolucci (2023)	Y	Y	N	N	N	Y	Y	Y	Y	Y	7
Örenler (2022)	N	N	Y	N	N	N	N	N	Y	Y	3
Cholewicki (2022)	Y	Y	Y	N	N	Y	Y	N	Y	Y	7
Hadamus (2021)	N	N	Y	N	N	N	Y	N	Y	N	3
Nadal-Nicolás (2020)	Y	Y	N	N	N	N	N	N	Y	Y	4
Castro Sánchez (2019)	Y	Y	N	N	N	Y	Y	Y	Y	Y	7
Moustafa (2015)	Y	Y	N	N	N	N	Y	Y	Y	Y	6
Nerbass (2010)	Y	N	Y	N	N	N	Y	N	Y	Y	5

[a]Item 1 (specification of eligibility of criteria), which relates to external validity, is not counted in the overall score, which ranges from 0 to 10.

Total PEDro scores of 0-3 are considered ‘poor’, 4-5 ‘fair’, 6-8 ‘good’, and 9-10 ‘excellent’.
